# Carotid Artery Stenting: A Single Center “Real World” Experience

**DOI:** 10.1371/journal.pone.0035300

**Published:** 2012-04-30

**Authors:** Nazmi Krasniqi, Michael Turgut, Marc Husmann, Marco Roffi, Urs Schwarz, Matthias Greutmann, Thomas F. Lüscher, Beatrice Amann, Roberto Corti

**Affiliations:** Cardiology, Cardiovascular Center, University Hospital Zurich, Switzerland; S.G. Battista Hospital, Italy

## Abstract

**Background:**

Percutaneous carotid artery stenting (CAS) became a widely used procedure in patients with symptomatic and asymptomatic carotid artery stenosis. However its role compared to carotid endarterectomy (CAD) remains questioned. We analysed the safety of carotid artery stenting program of a prospective CAS register program of a tertiary teaching hospital.

**Method:**

Between July 2003 and December 2010, 208 patients underwent CAS procedure. Baseline, procedural and follow-up data were prospectively collected. Primary peri-interventional outcome was defined as 30-day major adverse events (MAE), including death, stroke or myocardial infarction, and mid- to long-term follow-up outcome included ipsilateral stroke, myocardial infarction or death. Secondary outcome was restenosis rate ≥50% per lesion.

**Results:**

Unilateral carotid artery interventions were performed in 186 patients. In 22 patients CAS was performed bilaterally as stages procedures. The 30-day MAE rate was 1.9% consisting of two contralateral strokes and two ipsilateral stroke. Mean clinically follow-up was 22 months. Mid- to long-term MAE was 8.1% with 6.3% (n = 13) deaths, 1.9% (n = 4) myocardial infarctions and 0.9% (n = 2) ipsilateral stroke. The restenosis rate ≥50% per lesion was 4.3% at a mean follow-up of 22 months. Target lesion revascularization was performed in one patient, because of restenosis at 9 months follow-up after first CAS.

**Conclusion:**

Implementation of a carotid artery stenting program at a tertiary, teaching hospital is a safe method for treatment of carotid artery stenosis. The adverse event rate during mid-to-long-term follow-up suggests an appropriate patient selection.

## Introduction

Stroke is one of the leading causes of death following ischemic heart disease and is one of the most frequent reasons of permanent disability [1]. It has been estimated that significant stenosis of the internal carotid artery may be the predisposing condition in 5–12% of all strokes [2]. Carotid endarterectomy has been shown to reduce the risk of recurrent stroke by a half in patients with recent cerebrovascular symptoms associated with severe carotid stenosis [3]. Endovascular treatment of carotid stenosis by percutaneous transluminal balloon angioplasty or insertion of a stent is an accepted alternative to endarterectomy [4]. However, a meta-analysis (2007) found a higher risk of stroke or death within 30 days after endovascular treatment than after endarterectomy, and endarterectomy has remained the treatment of choice for carotid stenosis [5]. In addition, current results from the CAVATAS study show that restenosis is more common after endovascular treatment than after endarterectomy and is associated with recurrent ipsilateral cerebrovascular symptoms but that the risk of recurrent ipsilateral stroke is low [6].

In contrast, studies such as the Stenting and Angioplasty with Protection in Patients at High Risk for Endarterectomie trial (SAPPHIRE) suggested that CAS may have a better outcome than CEA in selected patients [7]. The SPACE trial (stent protected angioplasty versus carotid endarterectomy) failed to prove the non-inferiority of CAS for the 30-day complication rate but showed similar results at two years follow-up [8]. The EVA-3S trial (endarterectomy versus angioplasty in patients with severe symptomatic stenosis) even showed significant higher rates of death and stroke within 30 days in CAS compared to CEA [9]. In summary, all results from randomized trials the optimal role of carotid angioplasty/stenting versus endarterectomy remains unclear. While the EVA-3S trial has been criticized for the limited interventional experience requested for participation in the study [9], consensus on minimal training requirements and performance data of newly initiated CAS programs are lacking. Recently we reported on the safety of starting a carotid stenting program in 100 patients [10]. The present study aimed to further elucidate the efficacy and safety of carotid stenting program in a larger series of patients at a tertiary referral and teaching center.

## Methods

### Carotid Stenting Program and Patient Selection

Our registry included 229 consecutive patients who underwent carotid artery stenting (CAS) from July 2003 until December 2010. In 7 patients undergoing diagnostic angiography the revascularization procedure was not performed because of absence of severe stenosis (n = 5) and carotid artery occlusion (n = 2), respectively. In addition, in another 3 patients CAS was not performed because of severe vessel tortuosity and hence inaccessible carotid artery and thereafter referred for carotid endarterectomy. In all 10 patients the procedure was stopped without any neurologic or cardiovascular complications. 11 patients (5.3%) were excluded due to an incomplete follow-up, 208 patients remained for analysis. Patient characteristics are summarized in table 1. Interventional physician experience following a CAS-fellowship-training includes minimally 100 diagnostic cerebral angiographies and 40 CAS procedures performed as first or second operator during under supervision by an experienced interventional physician. The CAS interventions in our case series have been done by 2 Cardiologists with high volume caseload in coronary interventions. Their experience in working with 0.014” wires and guiding catheters is higher than optimal requested by guidelines. The caseload was each year higher, starting by 5 cases 2003, and ending by 48 cases 2010.

**Table 1:** Baseline Characteristics.

	n = 208
Age, gender	
Mean age, years	69
Range age, years	38–88
Age > 70 years, n (%)	65 (31)
male gender, n (%)	146 (70)
Cardiovascular risk factors, n (%)	
Diabetes mellitus	63 (30)
Dislipidämia	162 (78)
Hypertension	178 (86)
Current smoking	76 (36)
History of cardiovascular disease, n (%)	
Previous PCI	108 (52)
Previous CAGB	96 (46)
Previous myocardial infarction	64 (30)
Congestive heart failure	21 (10)
Previous CEA ipsilateral	16 (8)
Previous CEA contralateral	11 (5)
Previous cerebrovascular event or TIA	109 (52)
SAPPHIRE high risk characteristics, n (%)	
≥1 high risk features	92 (44)
≥2 high risk features	21 (10)
Carotid lesion characteristics, n (%)	n = 208
Symptomatic stenosis *	92 (44)
Contralateral occlusion	25 (12)
Contralateral stenosis ≥ 50%	77 (37)
Reccurent stenosis after endarterectomy	14 (7)

PCI; percutaneous coronary intervention; CABG; coronary artery bypass grafting.

CEA; carotid endarterectomy; TIA, transient ischemic attack.

SAPPHIRE; Stenting and Angioplasty with Protection in Patients at High Risk for Endarterectomy trial.

* TIA or CVI (cerebrovascular insult) in the preceding 6 months.

**Figure 1:**
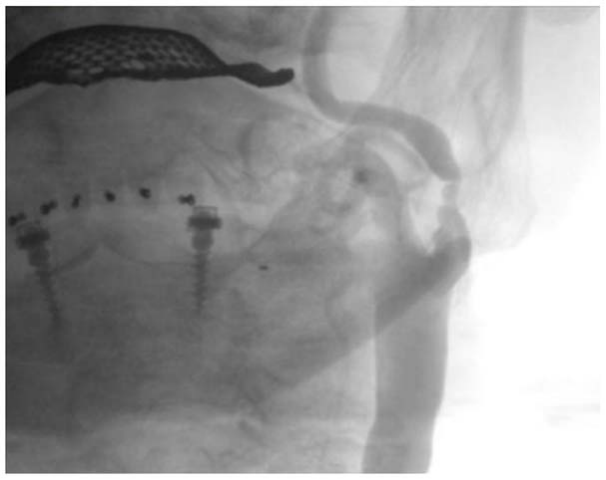
Conventional angiography of the left internal carotid artery with a severe stenosis by an 80 years old male patient.

Patients were considered for revascularization in the presence of a ≥70% asymptomatic or a ≥50% symptomatic stenosis of the internal carotid artery (Fig. 1).

Stenosis was considered symptomatic in the presence of transient ischemic attack or stroke affecting the corresponding territory in the preceding 6 months. Stenosis severity was assessed by color-coded duplexsonography (CCDS) and either confirmed by CT or MR angiography. In all patients a baseline imaging of the brain with CT or MR was performed. The indication for revascularization was approved by a neurologist in all cases (US). The neurologist examined all patients before and after the procedure. ECG and creatinin kinase (CK), CK-MB, and troponin were obtained systematically on admission and the day after CAS.

Written informed consent was obtained from all patients enrolled in the study. The present study was approved by the institutional review board (Cantonal Ethics Committee) of Zurich, Switzerland.

### Technique

**Figure 2:**
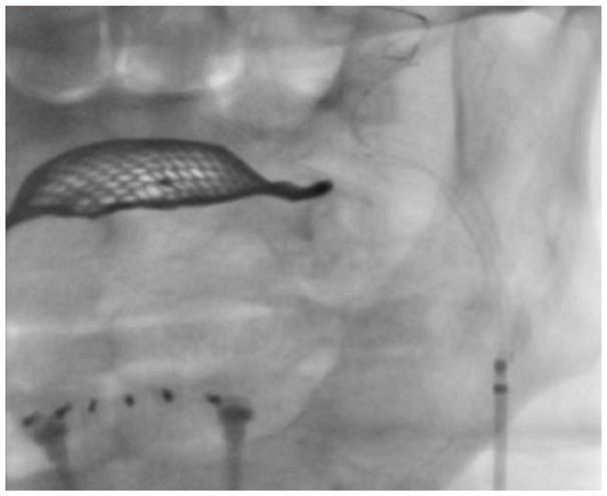
Catheterizing of the left common-and internal carotid artery and stent implantation.

All patients were pre-treated with aspirin and clopidogrel and during the procedure unfractionated heparin was administered to achieve an activated clotting time of 250–300 sec. Four-vessel angiography, consisting of at least a selective angiography of both common carotid arteries and a nonselective angiography of the subclavian and vertebral arteries, was performed using 5F diagnostic catheter unless contraindicated (e.g., in the presence of renal insufficiency, severe calcification or tortuosity of the aortic arch or the supraaortic vessels). Digital subtraction angiography of each vessel was obtained at the cervical and intracranial level. The stenting procedure was performed wih either a 8F guiding catheter advanced over a 125 cm-long 5F diagnostic catheter with telescoping technique or a 6F 90 cm-long sheath (Fig. 2).

**Figure 3:**
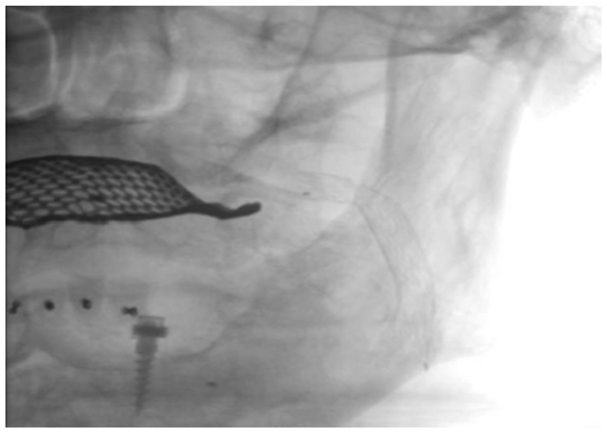
Stent implanted into the left internal carotid artery.

**Figure 4:**
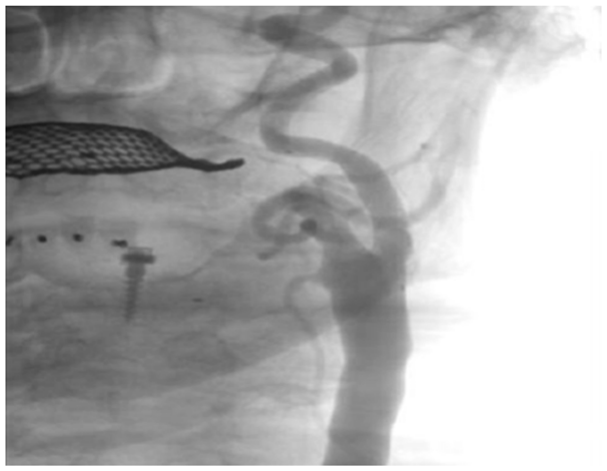
Angiogram after stenting the left internal carotid artery.

**Figure 5:**
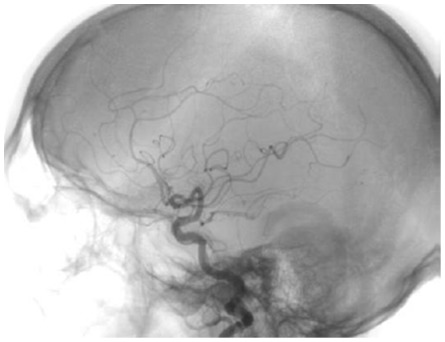
Intracranial control angiogram after procedure.

All procedures were attempted to be performed with proximal or distal protection devices that was at the discretion of the interventional physician and vascular conditions. To prevent bradycardia and hypotension, 0.5–1.0 mg of atropine was routinely administered intravenously prior to balloon inflation or stenting if no predilatation was performed. Following placement of the protection devices procedures were usually performed with predilation (3.5 mm to 4.5 mm), stent deployment, and postdilation (5.0 mm–6.5 mm). Choice of stent was at the discretion of the interventional physician (tapering, open versus closed cell design). Before retrieval of the protection device, final biplane angiogram of the stented lesion as well as intracranial views were obtained (Fig. 3,4,5).

### Outcome Measures

Follow-up consisted of CCDS and clinical examination at 1 month, 6 months, 12 months, and yearly thereafter. The primary outcome measures were in accordance to the Stenting and Angioplasty with Protection in Patients at High Risk for Endarterectomy (SAPPHIRE) trial. At 30 days, major adverse events (MAE) consisted of the composite occurrence of death, stroke, or myocardial infarction (MI). In addition, the rate of death, any stroke or myocardial infarction at follow-up was tracked. Minor stroke was defined as focal neurological deficit lasting more than 24 hr with Ranking score ≤2 and NIH stroke score ≤4, while major stroke was diagnosed in the presence of a Ranking score >3 or NIH score ≥15. Any rise in CK-MB or troponin or new pathological Q-waves on ECG defined MI. Restenosis was diagnosed in the presence of a ≥50% luminal narrowing as assessed by flow velocities using CCDS [10]. Technical success was defined by the coverage of the carotid lesion with a stent in the presence of a residual stenosis <50% and normal flow. Stenosis was classified as: not significant (0–49% stenosis), moderate (50–69%), severe (70–99%), or occluded (100%).

The peak systolic velocities of the common carotid and the internal carotid arteries and the end diastolic velocity of the internal carotid artery were recorded with CCDS.

### Statistics

Continuous variables were expressed as the mean±1 standard deviation. Categorical data were presented as actual numbers and percentages.

## Results

Unilateral carotid artery interventions were performed in 186 patients. Target lesion revascularization was performed in one patient, because of restenosis at 9 months follow-up after first CAS. Twenty one patients underwent bilateral staged CAS.

Detailed baseline characteristics of all patients with successful CAS are shown in Table 1.

Mean age was 69 years with a range age from 38–88 years (Table 1). Comorbidities were previous cardiac intervention in 100 patients (48%). This relatively high prevalence in cardiovascular comorbidities is shown in Table 1 in relation to the SAPPHIRE trial risk stratification with 1 or more high risk features in 54% of the cases. One third of the patients had a symptomatic carotid stenosis, 25 patients (12%) had a contralateral carotid artery occlusion and 14 patients (7%) had a recurrent carotid stenosis after CEA (Table 1).

Technical procedural success was achieved in all of the cases (Table 2). Emboli protection devices (EPD) were used in 99% of the procedures (included the 11 patients with balloon occlusion devices). In 1% no EPD were used due to severe tortuosities of the internal carotid artery. The lesions were predilated in 76% and postdilated in 97% of all procedures. One stent per procedure was used in 200 patients (96%). In 8 patients more than one stent were necessary to cover the long carotid lesions. In one patient, an additional stent was necessary to cover a dissection caused by catheter guiding.

**Table 2:** Procedural Data.

	n = 208
Angiographic and Doppler parameters	
Mean Angiographic degree of stenosis, % ^1^	83
Doppler flow velocities ^2^	
Vmax ICA systolic/diastolic, cm/sec	340/120
Vmax ICA/Vmax CCA	6
Drug regimen, n (%)	
Pretreatment with aspirin and clopidogrel ^3^	206 (99)
Atropin administration	184 (88)
Any periprocedural norepinephrine	62 (30)
Norepinephrine boluses	56 (26)
Norepinephrine drip at end of procedure	6 (3)
Unfractionated heparin	208 (100)
Technical characteristics, n (%)	
Procedural success	208 (100)
Emboli protection device use	206 (99)
Angioguard	86 (41)
Spider	19 (9)
Filterwire	59 (28)
Emboshield	33 (16)
Ballon occlusion device	11 (5)
Stents, n (%)	208 (100)
More than 1 stent	8 (4)
Type of stent	
Precise	132 (63)
Acculink, Braun, Herculink, Bigsize	11 (6)
Vivexx	20 (10)
Nexstent	7 (3)
Cristallo	28 (13)
Vascuflex	10 (5)
Postdilatation, n (%)	202 (97)

^1^ visual estimated.

^2^ Vmax; maximum velocity; ICA; internal carotid artery; CCA; common carotid artery.

^3^ One patient with aspirin allergy was treated periprocedurally with additional tirofiban and discharged on clopidogrel 150 mg/day for 1 month and 75 mg/day indefinitely.

During the CAS procedure in 26 patients (13%) occurred an internal carotid artery spasm which resolved either spontaneously or after administration of intra-arterial nitro-glycerine without any neurological complications (Table 3). Two patients had a seizure due to longer periode of hypotension during the intervention that spontaneously resolved. Periinterventional haemoglobin drop was observed in five patients without any postinterventional relevant bleeding at the access site, and most pro explainable source of blood loss. The facts that large volume of fluid were intravenously given during the procedure and the periinterventional blood loss through the catheters may be the reason of these anaemia. In one of these patients the anaemia was already known before. Seven patients with access groin hematomas were treated conservatively and no other access site complications were observed.

**Table 3:** Periprocedural Findings and Complications.

	n = 208
	n (%)
Internal carotid artery spasm	26 (13)
Seizure	2 (1)
Transfusion of erythrocytes	7 (3)
Femoral pseudoaneurysm, arteriovenousfistulas, dessection	0
Endovascular or surgical treatment offemoral access required	0

Major adverse event (MAE) rate within 30 days was 1.9%. Four patients suffered a stroke, two nonipsilateral. Two patients suffered an ipsilateral stroke, as the result of an air embolisation during balloon dilatation caused by a balloon defect. The contra lateral strokes were likely both due to periinterventional embolisation because of guiding catheter manipulation in the aortic arch. There were no deaths or MI within 30 days. There were 2.2% (2/92) and 0.8% (1/116) major adverse events in symptomatic and asymptomatic carotid artery stenosis, respectively.

The mean follow-up was 22 months with a follow-up range from 2–72 months. After 30 days of follow-up, 13 patients died (6.3%), 4 suffered a stroke (1.9%) and 4 had a MI (1.9%). The comorbidities of patients who died were: five patients with refractory heart failure, three with cancer (prostate, stomach, bottom lip), one who committed suicide, one with a rupture of an aortic aneurysm, one who died as consequence of his diseases (chronic heart- and kidney insufficiency, atrial fibrillation, and chronic obstructive pulmonary disease), and two in absence of an identifiable cause of death (one who suffered an intracranial bleeding six months before death and a coronary heart disease with chronic atrial fibrillation, six months after CAS, the other with Diabetes mellitus type II, a mild insufficiency of the mitral and aortal valve and a diastolic dysfunction with an ejection fraction of 62%, 23 months after CAS).

Two MI were in patients with a known coronary heart disease, 20 respectively 40 months after CAS. One suffered a MI during aorto-coronary bypass surgery six weeks after CAS and the fourth MI was observed 40 months post CAS in a patient with newly detected coronary heart disease manifested with an acute biventricular cardial decompenstation with dyspnea NYHA grade II and a progredient angina pectoris.

The overall MAE at follow-up was 8.2% (Table 4). The overall death or any stroke rate was 10%. The number of patients over 70 years was 65 (31%). The overall MAE rate in these patients was 13.8% (9 patients). The overall MAE rate in younger patients was 8.3% (12 patients).

**Table 4:** Major Adverse Events.

	n (%)
Within 30 days	208 Patients
Death	0
Stroke	4 (1.9)
Major ipsilateral	2 (0.9)
Major nonipsilateral	1 (0.5)
Minor ipsilateral	0
Minor nonipsilateral	1 (0.5)
Myocardial infarction	0
Death, Stroke or Myocardial infarction	4 (1.9)
After 30 days	208 Patients
Death	13 (6.3)
Stroke	4 (1.9)
Major ipsilateral	0
Major nonipsilateral	0
Minor ipsilateral	1 (0.5)
Minor nonipsilateral	3 (1.4)
Myocardial infarction	4 (1.9)
Overall	208 Patients
Death, Stroke or Myocardial infarction	17 (8.2)
at 30 days plus death or ipsilateral	
stroke within 31 days of follow-up	
Death or any Stroke	21 (10)
Overall (death or any stroke) Patients >70 years	9 (13.8%)

A CCDS 6 months post CAS were performed by 78% of all cases (164/208). The restenosis rate was 4.8% (9 patients with a restenosis ≥50%, respectively, and one patient with a stent occlusion). Target lesion revascularization rate was performed in one patient only due to an asymptomatic restenosis detected in 9 months follow-up with CCDS.

## Discussion

Our study demonstrates the short-and long-term safety of CAS regarding relevant end points performs well according to current recommendation guidelines.

All carotid stenosis could be successfully treated. In 99% of the procedures emboli protection devices (EPD) has been used without any complications. The benefit of EPDs has not been established in randomized controlled trials, and available data are conflicting [11]. In our trial the 30 days-stroke-rate by using EPDs is 1.9%, well aware of the causes of the four periinterventional strokes: two due to an air embolisation and two caused by guiding catheter embolisation in the aortic arch, means probably before the EPD could be installed.

The meta-analysis by Bonati et al [12] showed a higher risk for stroke or death in patients older than 70 years after carotid stenting within 120 days after randomization. In younger patients was the risk for adverse events similar in both groups. Another important point which was discussed in this meta-analysis was the experience of the interventional physicians, who performed the carotid stenting. The center recruitment rate of less than 1 patient was a predictor of adverse events.

Economopoulos et al. [13] confirmed in their meta-analysis the findings of Bonati et al. Another finding of this meta-analysis was the exhibition of lower rates of strokes and the higher rates of myocardial infarction in carotid endarterectomy than in carotid stenting patients.

The last published and one of the most important studies about carotid stenting, the CREST trial [14] showed no significant difference in the risk of the composite primary outcome of stroke, myocardial infarction or death between carotid artery stenting and carotid endarterectomy. The incidence of periprocedural stroke was higher in carotid stenting group than in endarterectomy group. On the other hand the incidence of periprocedural myocardial infarction was higher in endarterectomy group than in the stenting group. These findings confirm the findings of the meta-analysis by Economopoulos et al. They have an important clinical impact in the case of treating patients with primary cardiac disease, where the carotid stenting procedure may be preferred.

In our study the mean age of patients was 69 years. The number of patients over 70 years was 65 (31%). The overall MAE rate in these patients was 13.8% (9 patients). The overall MAE rate in younger patients was 8.3% (12 patients). These data correlate very well with the findings of both meta-analysis mentioned above.

The periprocedural rate of myocardial infarction was 0%, and the rate of stroke was 1.9%. These data confirm the finding of the CREST trial about the low periprocedural risk for myocardial infarction in carotid stenting group.

The MAE rate within 30 days was 1.9%, corresponding to four periinterventional strokes. Compared with the periprocedural death-and stroke-limits of 6% for symptomatic and 3% for asymptomatic lesions, for CEA set by the American Heart Association [15], the 30 days MAE-rate in our trial did definitively well. A meta-analysis showed 30 days MAE-rates after CAS up to 8% [16], which illustrates the more our good result. The overall ipsilateral stroke rate of only 1.4% and the restenosis rate of 4.8% at follow-up demonstrates well the safety of the procedure after long-term durability. Reported rates of early restenosis after CAS vary widely. Newly published long-term results concerning restenosis rate in the CAVATS study showed a three time higher incidence of developping a restenosis of ≥50% in endovascular than after endarterectomy [6]. In this trial the incidence of restenosis rate after endovascular treatment by balloon dilatation was significally higher than with a stent. From the 50 patients who received a stent, 23% of them after one year and 37% after five years, respectively, developed a restenosis of ≥50%, which is definitively higher than in our trial. This trial also demonstrated, that the greatest part of the restenosis occurred after one year follow-up in each surgical group, means that our restenosis rate result by a mean ultrasound follow-up of 22 months is well representative.

No periprocedural myocardial infarction was observed, even though cardiac heart enzymes and ECG were routinely controlled. That confirms the findings of SAPPHIRE trial, namely that the one-year-MAE- rate, means death, stroke and newly also MI included, was significantly lower in CAS than in CEA group, largely due to the higher incidence of perioperative MI in the CEA group [7]. That̀s an important point because of the facts that patients with a carotid stenosis have a higher prevalence of coronary artery disease despite the absence of cardiac symptoms [17].

The overall MAE-rate (8.2%) according to SAPPHIRE and the Death-or-any-Stroke-rate (10%) are at most driven by the higher incidence of death in comparison with the other MAE-factors but not correlating with long-term side effects or complications of CAS. The incidence of death is not surprising considered the mean age of 68 years and the high incidence of comorbidities.

In our institution cardiovascular events were allocated by a neurologist, who visited the patients before and after the intervention. An appropriate patient selection was one of the most important points. Only Patients with an adequate anatomy for percutaneous transcatheter approach qualified for the intervention. In elder patients we used the distal protection to reduce the risk of ipsilateral stroke. All these steps contributed to the overall risk reduction and safety of CAS.

The findings of this study are limited by the fact that this was a retrospective study. Furthermore, the study power is limited by the relatively small study population and shorter follow-up duration.

In summary, the results of this study suggest that CAS is a safe method for treatment of appropriate selected patients (especially patients younger than 70 years) with carotid stenosis, with good short-and-long-term results.

## References

[1] Murray CJ, Lopez AD (1997). Mortality by cause for eight regions of the world: Global Burden of Disease Study. Lancet.. 3;.

[2] Bates ER, Babb JD, Casey DE, Cates CU, Feldman TE (2007). ACCF/SCAI/SVMB/SIR/ASITN 2007 clinical expert consensus document on carotid stenting. J Am Coll Cardiol.. 2;.

[3] Barnett HJ, Taylor DW, Eliasziw M (1998). Benefit of carotid endarterectomy in patients with symptomatic moderate or severe stenosis. North American Symptomatic Carotid Endarterectomy Trial Collaborators. N Engl J Med.. 12;.

[4] Ricotta JJ, Malgor RD (2008). A review of the trials comparing carotid endarterectomy and carotid angioplasty and stenting. Perspect Vasc Surg Endovasc Ther..

[5] Luebke T, Aleksic M, Brunkwall J (2007). Meta-analysis of randomized trials comparing carotid endarterectomy and endovascular treatment. Eur J Vasc Endovasc Surg..

[6] Bonati LH, Ederle J, McCabe DJ, Dobson J, Featherstone RL (2009). Long-term risk of carotid restenosis in patients randomly assigned to endovascular treatment or endarterectomy in the Carotid and Vertebral Artery Transluminal Angioplasty Study (CAVATAS): long-term follow-up of a randomised trial. Lancet Neurol..

[7] Meyer SA, Gandhi CD, Johnson DM, Winn HR, Patel AB (2010). Outcomes of Carotid Artery Stenting in High-Risk Patients With Carotid Artery Stenosis: A Single Neurovascular Center Retrospective Review of 101 Consecutive Patients..

[8] Eckstein HH, Ringleb P, Allenberg JR, Berger J (2008). Results of the Stent-Protected Angioplasty versus Carotid Endarterectomy (SPACE) study to treat symptomatic stenoses at 2 years: a multinational, prospective, randomised trial.. Lancet.

[9] Mas JL, Trinquart L, Leys D, Albucher JF (2008). Endarterectomy Versus Angioplasty in Patients with Symptomatic Severe Carotid Stenosis (EVA-3S) trial: results up to 4 years from a randomised, multicentre trial. Lancet Neurol..

[10] Roffi M, Greutmann M, Eberli FR, Rainoni L, Lüscher TF (2008). Starting a carotid artery stenting program is safe. Catheter Cardiovasc Interv..

[11] Eskandari MK (2006). Design and development of mechanical embolic protection devices. Expert Rev Med Devices..

[12] Carotid Stenting Trialists’ Collaboration, Bonati LH, Dobson J, Algra A, Branchereau A (2010). Short-term outcome after stenting versus endarterectomy for symptomatic carotid stenosis: a preplanned meta-analysis of individual patient data. Lancet..

[13] Economopoulos KP, Sergentanis TN, Tsivgoulis G, Mariolis AD, Stefanadis C (2011). Carotid artery stenting versus carotid endarterectomy: a comprehensive meta-analysis of short-term and long-term outcomes. Stroke..

[14] Brott TG, Hobson RW, Howard G, Roubin GS, Clark WM (2010). Stenting versus endarterectomy for treatment of carotid-artery stenosis. N Engl J Med..

[15] Biller J, Feinberg WM, Castaldo JE, Whittermore AD, Harbaugh RE (1998). Guidelines for carotid endarterectomy: a statement for healthcare professionals from a Special Writing Group of the Stroke Council, American Heart Association. Circulation..

[16] Zahn R, Hochadel M, Grau A, Senges J (2005). Stent-supported angioplasty versus endarterectomy for carotid artery stenosis: evidence from current randomized trials. Z Kardiol..

[17] Hofmann R, Kypta A, Steinwender C, Kerschner K, Grund M (2005). Coronary angiography in patients undergoing carotid artery stenting shows a high incidence of significant coronary artery disease. Heart..

