# Correction: Carotid Artery Stenting: A Single Center “Real World” Experience

**DOI:** 10.1371/annotation/42028902-fdcd-4e2c-9ec0-62f01edc30fa

**Published:** 2012-08-24

**Authors:** Nazmi Krasniqi, Michael Turgut, Marc Husmann, Marco Roffi, Urs Schwarz, Matthias Greutmann, Thomas F. Lüscher, Beatrice Amann-Vesti, Roberto Corti

The ninth author's name was spelled incorrectly. The correct name is: Beatrice Amann-Vesti.

Information was missing from the affiliation. The correct affiliation is: Cardiology, Angiology, and Neurology, Cardiovascular Center, University Hospital Zurich, Switzerland 

